# Release of ATP from Marginal Cells in the Cochlea of Neonatal Rats Can Be Induced by Changes in Extracellular and Intracellular Ion Concentrations

**DOI:** 10.1371/journal.pone.0047124

**Published:** 2012-10-10

**Authors:** Yating Peng, Jie Chen, Shan He, Jun Yang, Hao Wu

**Affiliations:** 1 Department of Otolaryngology-Head and Neck Surgery, Xinhua Hospital, Shanghai Jiaotong University School of Medicine, Shanghai, China; 2 Ear Institute, Shanghai Jiaotong University School of Medicine, Shanghai, China; 3 Department of Otolaryngology, Integrated Chinese and Western Medicine Hospital of Zhejiang Province, Hangzhou Red Cross Hospital, Hangzhou, China; 4 Department of Otolaryngology, Shanghai Children's Medical Center, Shanghai, China; 5 Department of Otolaryngology, People's Hospital of Shanghai Pudong District, Shanghai, China; Universitat Pompeu Fabra, Spain

## Abstract

**Background:**

Adenosine triphosphate (ATP) plays an important role in the cochlea. However, the source of ATP and the mechanism by which it is released remain unclear. This study investigates the presence and release mechanism of ATP in vitro cultured marginal cells isolated from the stria vascularis of the cochlea in neonatal rats.

**Methods:**

Sprague-Dawley rats aged 1–3 days old were used for isolation, *in vitro* culture, and purification of marginal cells. Cultured marginal cells were verified by flow cytometry. Vesicles containing ATP in these cells were identified by fluorescence staining. The bioluminescence assay was used for determination of ATP concentration in the extracellular fluid released by marginal cells. Assays for ATP concentration were performed when the ATP metabolism of cells was influenced, and ionic concentrations in intracellular and extracellular fluid were found to change.

**Results:**

Evaluation of cultured marginal cells with flow cytometry revealed the percentage of fluorescently-labeled cells as 92.9% and 81.9%, for cytokeratin and vimentin, respectively. Quinacrine staining under fluorescence microscopy revealed numerous green, star-like spots in the cytoplasm of these cells. The release of ATP from marginal cells was influenced by changes in the concentration of intracellular and extracellular ions, namely extracellular K^+^ and intra- and extracellular Ca^2+^. Furthermore, changes in the concentration of intracellular Ca^2+^ induced by the inhibition of the phospholipase signaling pathway also influence the release of ATP from marginal cells.

**Conclusion:**

We confirmed the presence and release of ATP from marginal cells of the stria vascularis. This is the first study to demonstrate that the release of ATP from such cells is associated with the state of the calcium pump, K^+^ channel, and activity of enzymes related to the phosphoinositide signaling pathway, such as adenylate cyclase, phospholipase C, and phospholipase A_2_.

## Introduction

Adenosine triphosphate (ATP) is a key signaling molecule in the cochlea, where it regulates sound transduction, hearing sensitivity, the active mechanical amplification by outer hair cells (OHCs), cochlear potential, cochlear homeostasis, and vascular tension [1]–[3]. Reportedly, when ATP is released from an intracellular source it displays features of a fast-acting intercellular messenger, such as the following: (1) release in a controllable pattern; (2) ligand-specific transduction coupling between the membrane receptor and signals conducted; and (3) rapid degradation for termination of the reaction [4]. ATP receptors are widely distributed in the cochlea. For example, P_2_X receptors, which are ionotropic and constitute a Ca^2+^ channel, are present on hair cells, spiral ganglion cells, Deiters' cells, and the epithelial cells of the Reissner's membrane. Similarly, P_2_Y receptors, which are G-protein coupled receptors and thus elicit their effects through phospholipase C (PLC) to either release intracellular Ca^2+^ or activate adenylate cyclase, are present in hair cells and marginal cells of the stria vascularis [5]–[7]. Altogether, this makes ATP an important candidate neurotransmitter for afferent nerves in the cochlea. While many functions of ATP in the cochlea are being revealed, its sources and release mechanism remain unclear.

While examining the mechanism of the release of ATP in the cochlea, Zhao et al. found that the hemichannel of gap junctions might mediate the release of ATP from supporting cells [8]. Gap junctions are a type of cytoplasmic conduit that allows the passage of small molecules, such as metabolites and signaling molecules. Each gap junction is composed of two hemichannels, each of which is made up of six connexin subunits. In the cochlea, the connexin of gap junctions is expressed only on supporting cells, not on hair cells. The gap junctions in the cochlea might play an important role in intercellular signaling and metabolite exchange [9]. However, the issue of which kind of supporting cells releases and stores ATP, remains unclear. In this regard, Housley et al. suggested that inner hair cells (IHCs) and OHCs might release ATP and glutamate by synergistic mechanisms, thus contributing to an ATP source in the perilymph [5]. In turn, this would suggest that hearing codes may be regulated by synapses between spiral ganglion cells and IHCs or OHCs through the P_2_X_2_ and P_2_X_7_ receptor subunits, such as ion-gated channels mediated by ATP [5]. Along these lines, Wangemann et al [10] observed Ca^2+^-dependent release of ATP in the organ of Corti. Increasing Ca^2+^ concentrations activated more ATP-releasing channels, further facilitating the spread of calcium waves. Results suggested that the release of ATP from hair cells is dependent upon storage of free Ca^2+^ in the cytoplasm, but there is no direct evidence supporting the conclusion that ATP can be released from hair cells. Therefore, other potential sources of ATP in the cochlea have been proposed. For instance, the Kölliker organ is a temporary structure that exists in the developing mammalian cochlea. It consists of columnar supporting cells [11], [12]. In this regard, the release of ATP from these cells in neonatal SD rats was demonstrated by means of the patch clamp technique [13]. Similarly, Zhao et al. demonstrated that ATP was released into the perilymph through hemichannels in supporting cells in the Corti's organ of the mature cochlea. In turn, ATP acted through P_2_ purinergic receptors on the lateral surface of OHCs, reducing the electromotility and voltage-dependence of these cells [8]. Together, these studies suggest the existence of a purinergic signal pathway between supporting cells and hair cells within the cochlea, which may control hearing sensitivity.

Further evidence supports the notion that ATP is released into the endolymph by marginal cells in the stria vascularis of the cochlea. For instance, purinergic compounds in the form of vesicles have been identified in marginal cells of the cochlea in guinea pigs, as observed under laser confocal microscopy, transmission electron microscopy, and quinacrine immunofluorescence staining [14]. The variations in size and shape of these vesicles was consistent with transport of purinergic compounds across the cell membranes of marginal cells [14]. This may suggest a purinergic exchange between marginal cells and the endolymph, with ATP being released into the scala media through following an exocytotic mechanism.

The stria vascularis, located on the lateral wall of the cochlea, consists of three layers of cells, basal, middle, and marginal. The most important layer is the one formed by irregular epithelial cells, whose diameters range from 10 to 15 µm [14]. Marginal cells are one type of irregular epithelial cell. High concentrations of K^+^ can be generated by basal and middle cells in the stria vascularis. A Na^+^-K^+^-2Cl^−^ transporter then transfers K^+^ into marginal cells, where it eventually diffuses into the endolymph in the scala media along an electrochemical gradient. The accumulation of high concentrations of K^+^ in the endolymph and circulation of K^+^ between the endolymph and perilymph are the basis for the maintenance of function and homeostasis in the cochlea. Current knowledge supports the notion that the ATP released by marginal cells regulates homeostasis by increasing the flow of K^+^ out of the scala media and decreasing G-protein mediated flow of K^+^ into the endolymph. For instance, this mechanism is postulated to decrease cochlear sensitivity to external noise [5]. In addition, an influence of ATP upon K^+^ transportation in marginal cells was also reported [15]. In this regard, Enkvetchakul et al. found that ATP acts as an allosteric regulatory molecule, controlling the opening and closing of K^+^ channels and therefore the outflow of K^+^ and ultimately the K^+^ concentration in the endolymph [16]. Indirect support for the idea that marginal cells are the source of the ATP involved in the regulation of these events has been provided by evidence of the presence of ATP vesicles in the lateral wall of the cochlea [17]. The presence of these ATP-containing vesicles on marginal cells of the stria vascularis was determined via quinacrine, a stain that fluoresces when specifically bound to high concentrations of ATP [14], [18]. In this study, marginal cells from the stria vascularis of neonatal rats were successfully isolated, cultured, and purified. These cells were verified using surface fluorescent markers and flow cytometry. Vesicles containing ATP in these cells were identified by fluorescence staining. This is the first time that the ATP released into the extracellular fluid from marginal cells has been measured using bioluminescence assays. Notably, the amount of ATP released from marginal cells was found to be influenced by intracellular and extracellular ionic concentrations, such as those of K^+^ and Ca^2+^. Alteration of the concentration of intracellular Ca^2+^ induced by inhibition of the phosphoinositide signaling pathway also influenced the release of ATP from these cells.

## Results

### Verification of marginal cells by flow cytometry

We first isolated and cultured marginal cells from the neonatal rat cochleas. The stria vascularis and spiral ligament were dissected and dissociated as single cells. The marginal cells were purified by subculturing the primary cells for 2 weeks. The culture medium was refreshed every 3 days. Previous study confirmed the epithelial origin of cultured marginal cells in the stria vascularis by the expression of cytokeratin and vimentin [19]. We examined the purity of the cultured marginal cells using cytokeratin and vimentin antibodies. Flow cytometry revealed the percentage of cytokeratin and vimentin positive cells were 92.9% and 81.9% (figures not shown) respectively. Therefore, these results were consistent with a pure culture of marginal cells.

### Identification of vesicles containing ATP in cultured marginal cells with quinacrine

**Figure 1:**
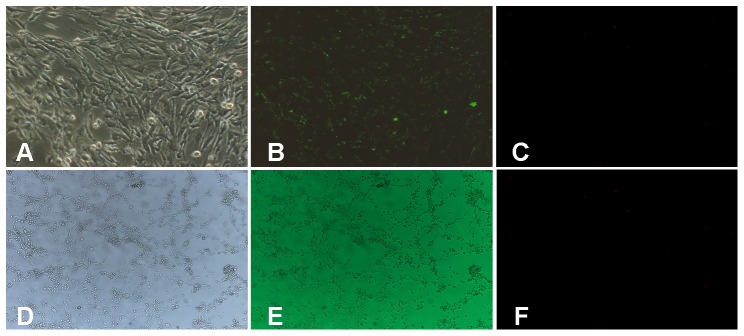
Morphological identification of cultured marginal cells and 3T3 cells. **A**, Under phase contrast microscopy, marginal cells were arranged like paving stones, and displaying a polygonal shape and large nuclei (×100). **B**, Under fluorescence microscopy, numerous star-like green pots were observed within the cytoplasm (×100). **C**, Negative control of marginal cells (background fluorescence). **D**, Under phase contrast microscopy, 3T3 cells were arranged spindle -shaped flat structure with cell protrusions and large nuclei(×40). **E**, Under fluorescence microscopy, no positive green staining was observed within the cytoplasm (×40). **F**, Negative control of 3T3 cells (background fluorescence).

Quinacrine is an acridine derivative that binds specifically to adenine-related substances and emits fluorenscence [18]. Previous studies have shown that this chemical can be used to identify vesicles containing ATP in marginal cells [14]. Under phase contrast, purified marginal cells presented as polygonal cobblestones with large nuclei, arranged in single, polar layer on the culture plate (Fig. 1A; corresponds to 21-days in culture), 3T3 cells were arranged spindle -shaped flat structure with cell protrusions and large nuclei and were fully attached to culture dish (Fig. 1D, corresponds to 7-days in culture) When quinacrine was added to the cultured marginal cells, numerous star-like green spots was observed in the cytoplasm of marginal cells. No such staining was observed in the cell nuclei (Fig. 1B). No positive green staining was observed within the cytoplasm of 3T3 cells (Fig. 1E). Fig. 3C and 3F are negative controls of marginal cells and 3T3 cells (background fluorescence), respectively. This suggested ATP containing vesicles were present in marginal cells. Next we started to examine the factors that control the ATP release from marginal cells.

### Quantification of ATP using bioluminance

**Figure 2:**
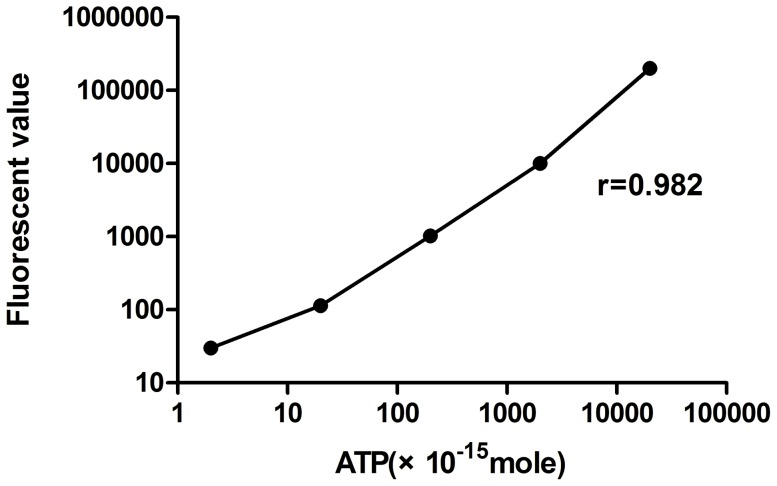
ATP standard curve. A strong linear correlation between ATP concentrations and fluorescence values is shown.

To quantitatively investigate the ATP release from the marginal cells, we measured its concentration through bioluminescence. A 10-fold serial dilution of the ATP standard was used to measure the fluorescence with CellTiter-Glo luminescent. Bioluminescence was read using a black 96-well plate to avoid optical cross-talk. The concentrations of ATP solution was plotted according to the fluorescent values and were used as a standard curve. The fluorescence values from five serial concentration of ATP are shown in Fig. 2. A strong linear relationship between ATP concentrations and fluorescence levels was observed with a R value of 0.9826. To minimize the experimental variations, a standard curve was measured in each experiment and the corresponding concentration of ATP was calculated.

### Influence of ATP metabolism on ATP release from marginal cells

**Figure 3:**
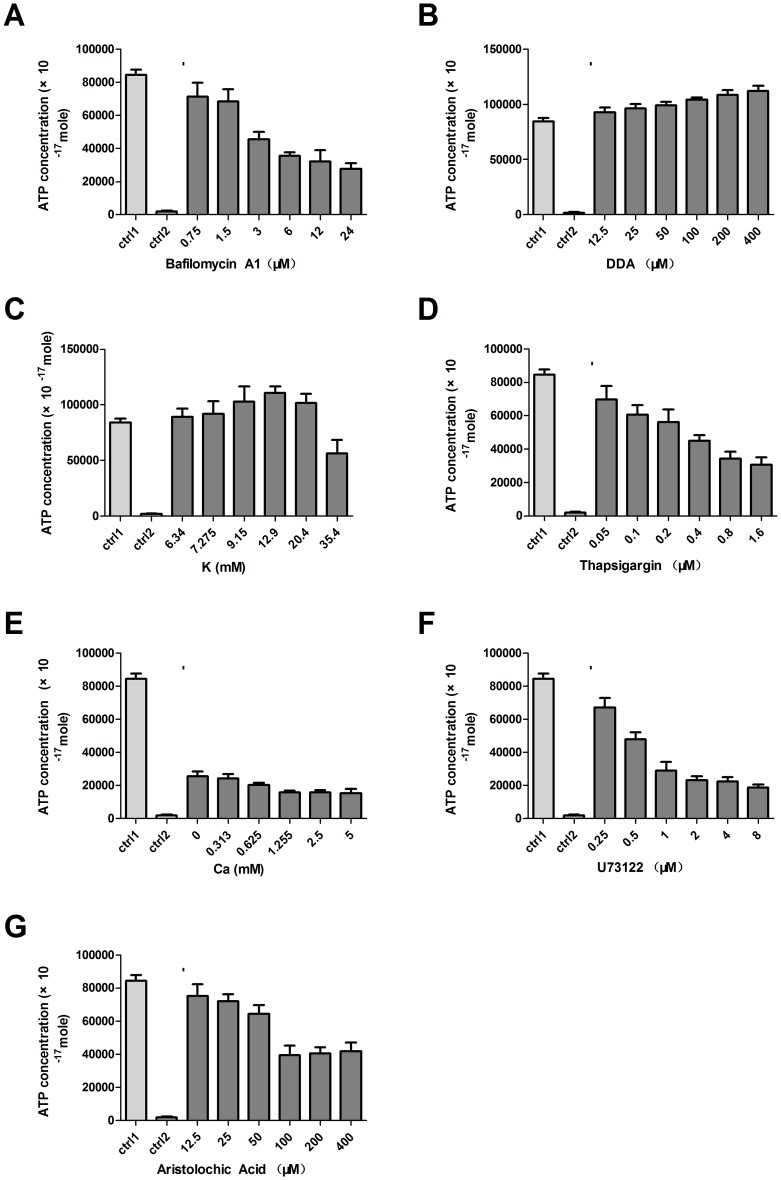
ATP release from marginal cells. **A**. Bafilomycin A1, a specific inhibitor of vacuolar-type H^+^-ATPase, inhibited the release of ATP from marginal cells. **B**. DDA, a specific inhibitor of adenylate cyclase, increased the release of ATP from marginal cells. **C**. Extracellular K^+^ concentration increased the release of ATP from marginal cells. **D**. Influence of thapsigargin, which decreases the intracellular Ca^2+^ concentrations inhibited the release of ATP from marginal cells. **E**. Extracellular Ca^2+^ concentrations inhibited the release of ATP from marginal cells. **F**. U73122 which affects the release of Ca^2+^ from intracellular stores inhibited the ATP releasing from marginal cells. **G**. Aristolochic acid, a PLA_2_ inhibitor decreased the release of ATP from marginal cells. The release of ATP was significantly changed after each treatment (n = 8, P<0.05).

Bafilomycin A_1_, a specific inhibitor of vacuolar-type H^+^-ATPase was added to marginal cell suspensions in order to test whether this was responsible for ATP transport across the cell membrane [20], [21]. As hypothesized, the concentrations of ATP in the extracellular fluid significantly and gradually decreased along with increasing concentrations of bafilomycin A1 (Fig. 3A). The ATP released from the cultured marginal cells without the addition of bafilomycin A1 was used as a positive control (ctrl1). 3T3 cells, a fibroblast cell line in which ATP vesicles were not present was used as a negative control (ctrl2) with the addition of test reagents. ATP concentrations differed significantly between the test cells and the control groups (n = 8, *P*<0.05, paired *t* test).

Didecyl adipate (DDA), a specific inhibitor of adenylate cyclase, was added to marginal cell suspensions in order to confirm it was responsible for ATP metabolism because ATP can be converted into cAMP by adenylate cyclase [22], [23]. The concentrations of ATP in the extracellular fluid increased as DDA was added to marginal cell suspensions (Fig. 3B). The same controls were used as in bafilomycin A1 experiments. The concentrations of ATP in the test cells and the control groups differed significantly (n = 8, *P*<0.05, paired *t* test).

### Mechanism of the release of ATP from marginal cells

#### Influence of extracellular K^+^ concentration on the release of ATP from marginal cells

Previous studies have shown that ATP can interact with the open state of the K_ATP_ channel, so K^+^ was added to marginal cell suspensions in order to determine the effect of intracellular K^+^ upon ATP release [16]. Initially, ATP concentrations increased as the concentration of extracellular K^+^ increased, with ATP levels peaking at 110634.28±6043.70×10^−17^ M, with a K^+^ concentration of 12.9 mM. At higher K^+^ concentrations, ATP concentrations decreased (Fig. 3C). ATP concentrations differed significantly between the test cells and the control groups of the cells without the addition of K^+^ and the 3T3 cells with the addition of K^+^ (n = 8, *P*<0.05, paired *t* test). It should be noted that the extracellular concentration of K^+^ was calculated as the sum of the added of K^+^, i.e. 0 to 30 mM and the 5.4 mM K^+^ from the RPMI-1640 culture medium.

#### Influence of intracellular and extracellular Ca^2+^ concentrations on ATP release from marginal cells

Thapsigargin, which is known to decrease the intracellular Ca^2+^ concentrations, was used to determine the effect of intracellular Ca^2+^ upon the release of ATP [24]. With the addition of increasing concentrations of thapsigargin to the marginal cells, the release of ATP was significantly decreased (Fig. 3D). As expected, ATP concentrations differed significantly between the test cells and the control groups of the cells without thapsigargin or no ATP vesicle containing 3T3 cells with thapsigargin (*P*<0.05, paired *t* test). Mechanically induced intercellular calcium waves can be inhibited by depleting intracellular calcium stores with thapsigargin [25] and can be elicited in the absence of extracellular calcium [26]. The concentration of extracellular Ca^2+^ in marginal cell suspensions was changed in order to determine the effects of extracellular Ca^2+^ upon ATP release. Even when extracellular Ca^2+^ was completely chelated with EGTA, marginal cells continued to release ATP. As the concentration of extracellular Ca^2+^ increased, the release of ATP decreased. However, the amount of ATP released remained at baseline when the extracellular concentration of Ca^2+^ reached 1.25 mM or above (Fig. 3E). Concentrations of ATP differed significantly between test cells and the control groups as used above (n = 8, *P*<0.05, paired *t* test).

#### Influence of intracellular 1,4,5-inositol trisphosphate (IP_3_) on the release of ATP from marginal cells

The PLC inhibitor U73122 was used to affect the release of Ca^2+^ from intracellular stores via the phosphoinositide pathway [27]. When concentrations of U73122 remained within the range of 0.25 to 8 µM, as U73122 increased, the release of ATP decreased (Fig. 3F). This suggested that reducing IP3 synthesis through phospolipidase C inhibition could also cause a reduction in the endoplasmic reticulum Ca^2+^ stores and a significant concomitant reduction in the release of ATP. The concentration of ATP differed significantly between test cells and the control groups as used above (n = 8, *P*<0.05, paired *t* test).

#### Influence of phospholipase A_2_ (PLA_2_) on the release of ATP from marginal cells

Aristolochic acid is a plant derivative reported to inhibit PLA_2_ [28]. PLA_2_ hydrolyzes phospholipids to release arachidonic acid and activate PLC. PLC will increase the intracellular release of Ca^2+^ and in turn the extracellular release of ATP. When concentrations of aristolochic acid ranging from 100 to 400 µM were added to the marginal cell suspension, the release of ATP was significantly decreased. However, with concentrations of aristolochic acid less than 100 µM, the release of ATP did not tend to differ significantly from that of the control groups(Fig. 3G). The controls are the test cells without addition of aristolochic acid (ctrl1) or 3T3 cells with aristolochic acid (ctrl2). These changes were all statistically significant (n = 8, *P*<0.05, paired *t* test).

## Discussion

Current knowledge supports the notion that marginal cells in the stria vascularis of the cochlea are an important source of ATP within this organ. Friedmann et al. demonstrated the existence of exocytosis in marginal cells, and it was proposed that the vesicular structures observed in those cells might be involved in the exchange of substances in the endolymph of the scala media [29]. Another group further reported not only widespread pinocytotic action on the surface of marginal cells but also a specific display of quinacrine fluorescence, suggesting that high concentrations of ATP are carried within these vesicles [14]. On a stretched preparation of the entire membranous labyrinth, specific quinacrine staining was observed only in the region of the stria vascularis [14]. The mechanism by which ATP is released from these cells is still poorly understood.

In the present study, we isolated and puried marginal cells in which epithelium cell specific markes, cytokeratin and vimentin were expressed. We then used this culture to further probe the factors controlling the release of ATP from these cells.

The mechanism by which ATP crosses cell membranes has also been studied in other cell systems. Using immunofluorescence staining and total-internal-reflection fluorescence microscopy (TIRFM), Zhang et al. demonstrated the presence of ATP-rich lysosomes in astrocytes and showed that ATP was released by a kiss-and-out exocytotic mechanism [30]. Considering the challenges involved in performing *in vivo* studies on the marginal cells of the stria vascularis, Muñoz et al. were only able to measure ATP in vesicular fractions extracted from the lateral wall of the cochlea and organ of Corti. Their measurements included a determination of free and total ATP using luciferase enzyme assays to calculate the amount of ATP captured in vesicles [17]. While these researchers were able to demonstrate the presence of large amounts of ATP in the vesicles of the stria vascularis, there is no direct evidence pointing at marginal cells as the exact origin of such vesicles. In this study, staining of a pure culture of marginal cells with quinacrine revealed numerous green, star-like spots located within the cytoplasm. However, such green staining was not observed in 3T3 cells. Therefore, 3T3 cells were used as a control in our study. This is consistent with previous findings and supports the notion of ATP vesicles originating from marginal cells of the stria vascularis [14].

We then used a bioluminescence assay to measure the fluorescence value of ATP in extracellular fluid of marginal cells and 3T3 cells. The ATP standard curve generated in this way showed concentrations of ATP within the range of 1×10^−15^M to 100,000×10^−15^M to be linearly correlated to fluorescent values. This allowed for a quantitative measurement of ATP concentrations. This method was found to be more sensitive, precise, and consistent than the fluorescein/luciferase assays previously used on vesicles extracted from tissue homogenates [17]. To further substantiate the ability to measure ATP released from marginal cells into the extracellular fluid, experiments were conducted involving bafilomycin A1 and DDA. Bafilomycin A1 is a selective inhibitor of voltage dependent (v-) ATPases [20], [21]. These are responsible for maintaining an electrochemical proton gradient for reuptake of ATP through secretory granules. This is required for ATP synthesis. In this study, the concentration of extracellular ATP decreased with the addition of bafilomycin A1. Similarly, the release of ATP into extracellular fluid involves hydrolysis of ATP to ADP by adenosine phosphatase, located on the cell surface. ATP can also be converted into cAMP by adenylate cyclase. When DDA, which is a specific inhibitor of adenylate cyclase [31], was added to the culture medium, the production of cAMP decreased as the concentration of ATP increased. Our experiments indicated that these effects were specific of marginal cells when compared to the effects on 3T3 cells in which no ATP vesicles were present. Together, these results confirm the specificity of the ATP detection system used in this study, which can therefore be said to be a reliable system for further elucidation of the effects of the release of ATP from the marginal cells of the stria vascularis.

The stria vascularis is the metabolically active epithelial tissue of the cochlea, and as such it regulates electrolyte exchange with the endolymph, generating a positive intracochlear potential. High concentrations of K^+^ are generated by basal and intermediate cells in the stria vascularis. This K^+^ can be transported to marginal cells through Na^+^-K^+^-2Cl^−^ channels. It then diffuses into the endolymph through K^+^ channels that open along the electrochemical gradient [32]. High concentrations of K^+^ in the endolymph and positive intracochlear potential are vital for the mechano-electrical transduction of hair cells because K^+^ can pass through the mechanical-gated channel located at the second and third row's stereocilia, as indicated by the large gap in potential between the endolymph and IHCs and OHCs [5]. In this study, the ATP released by marginal cells was dependent on the concentration of extracellular K^+^. In this way, ATP concentrations increased alongside K^+^, and peaked at a K^+^ concentration of 9.15 mM, after which the release of ATP decreased as the concentration of K^+^ increased. These results further support the idea that marginal cells take part in the regulation of K^+^ metabolism, especially because the stria vascularis faces the endolymph, where high concentrations of K^+^ are necessary for normal cochlear function. Others researchers have also postulated that one of the functions of ATP released by marginal cells in the stria vascularis is the regulation of homeostasis through increases in K^+^ outflow from the scala media, decreases in G-protein mediated K^+^ flow into the endolymph, and ultimately decreasing the sensitivity of the cochlea to noise [3], [5]. Through polymerase chain reaction and patch clamp experiments, Enkvetchakul et al. concluded that ATP regulates the opening and closing of K^+^ channels in an allosteric manner [16]. We postulate that this type of mechanism may operate in the marginal cells of the stria vascularis, controlling outflow of K^+^ and, indirectly, the concentration of K^+^ in the endolymph.

Understanding the mechanisms regulating the release of ATP release from the stria vascularis is also an important prelude in further understanding cochlear physiology. In this regard, Suzuki et al. suggested that the release of ATP from these cells caused an increase in intracellular Ca^2+^ from intracellular stores, which did not require the presence of extracellular Ca^2+^ [33]. Thapsigargin is an inhibitor of the Ca^2+^-ATP enzyme, responsible for replenishing Ca^2+^ into the endoplasmic reticulum. In this way, inhibition of this pump decreases the intracellular concentration of Ca^2+^ [24]. In this study, the concentration of ATP was gradually but significantly decreased as the concentration of thapsigargin increased, corroborating close relationship between intracellular Ca^2+^ concentrations and the release of ATP from marginal cells. A previous report similarly showed that marginal cells responded to ATP with a slight but significant increase in the intracellular concentration of Ca^2+^, independent of that of extracellular Ca^2+^ [33]. This suggested that intracellular stores were the source of Ca^2+^. We found that when extracellular Ca^2+^ concentrations were between 0 and 1.25 mM, the concentrations of ATP released from marginal cells were negatively correlated with those of extracellular Ca^2+^. However, when the concentration of extracellular Ca^2+^ reached 1.25 mM, the release of ATP remained stable regardless of further changes in the concentration of Ca^2+^. Similarly, Zhao et al. found that the quantity of ATP released through connexin hemichannels in the cochlea of guinea pigs could be finely modulated by changes in extracellular Ca^2+^ concentrations within well-defined ranges [8]. Since our results are consistent with those of previous reports, we speculated that the release of Ca^2+^ from intracellular stores plays a major role in the release of ATP from marginal cells. Extracellular Ca^2+^ may modulate this response within certain ranges in order to maintain overall tissue homeostasis.

As part of the objectives set to ascertain the molecular pathways involved in the release of ATP from marginal cells in the stria vascularis, we next set to investigate the phosphoinositide pathway. Activation of PLC leads to cleavage of phosphatidylinositol 4, 5-bisphosphate (PIP_2_) and production of diacylglycerol (DAG) and IP_3_, the latter which binds to its receptors in intracellular stores to elicit Ca^2+^ release. In turn, intracellular Ca^2+^ and DAG activate PLA_2_, which hydrolyzes phospholipids to release arachidonic acid. This may then activate PLC [34]–[36]. Interactions between phospholipase and Ca^2+^ messenger form a complicated signal transduction network; for instance, PLC, once activated, allows the release of Ca^2+^ stored within the cell. Aristolochic acid is an antagonist of PLA_2_ [28]. In this study, we hypothesized that inhibition of PLC with U73122 would significantly reduce the amount of Ca^2+^ released from intracellular stores, thereby significantly reducing the release of ATP. Using rabbit epithelial cells of the respiratory system Sanderson et al. found that the release of ATP could be blocked by U73122 [37]. Similarly, in the present study, increasing the concentration of U73122 from 0.25 to 1 µM resulted in a significant decreases in the concentration of ATP. Thus precluding IP3 synthesis via inhibition of PLC, caused a decrease in the release of Ca^2+^ from the endoplasmic reticulum, ultimately decreasing the concentration of ATP. Once the concentrations of U73122 and aristolochic acid exceeded a certain threshold the concentration of ATP did not decline further.

In conclusion, this is the first study in which the release of ATP from marginal cells in the stria vascularis of the cochlea was measured using a bioluminescence assay. Our results confirmed beyond any doubt the presence and release of ATP from cultured marginal cells. Notably, we provide novel evidence regarding the mechanism by which ATP is released from these cells. In this way, the release of ATP from marginal cells was influenced by changes in concentration of intracellular and extracellular ions, namely extracellular K^+^ and intra- and extracellular Ca^2+^. This is also the first study to demonstrate that the release of ATP is associated with the state of the calcium pump, K^+^ channel, and activity of enzymes related to the phosphoinositide signaling pathway, such as adenylate cyclase, PLC, and PLA_2_. Future studies may be aimed at elucidating the regulating pathway and mechanism of the release of ATP from marginal cells *in vivo*.

## Materials and Methods

### Experimental animals, cells and reagents

#### Experimental animals

Sprague-Dawley rats of both sexes, 1–3 days old, were provided by the Shanghai Laboratory Animal Center of the Chinese Academy of Sciences. All parents of these neonatal rats were tested for positive auricle reflexes. In this study, animals were used in strict accordance with protocols approved by the Animal Use and Care Committee of Shanghai Jiaotong University School of Medicine (License No. (Shanghai): 2008-0052).

#### Experimental cells

3T3 cells, a fibroblast cell line were purchased from America type Culture Collection(ATCC) (Manassa, VA, U.S) and were used as a negative control in our study (ctrl_2_). 3T3 cells are similar to fibroblast cells existing in the cochlea of neonatal rats.

#### Reagents

Collagenase (type I), PBS (1×), RPMI-1640 medium and DMEM medium, 10% fetal bovine serum (FBS, vol/vol), 0.1% antibiotics (containing 1000 U/mL penicillin and 10 mg/mL streptomycin), 0.25% pancrease-0.02% EDTA mixture (containing 0.25% trypsin w/vol and 0.02 EDTA w/vol) were purchased from GIbco Company and CellTiter-Glo™ kits were from Promega Company. Anti-cytokeratin IgG, anti-vimentin IgG, FITC-anti-rabbit IgG, quinacrine solution (1×10^−6^ mol/L), chelerythrine chloride, aristolochic acid, bafilomycin A1, DDA, and heparin sodium were purchased from Sigma Chemical Company and U73122 was from Wako Pure Chemical Inc. Thapsigargin was purchased from Alomone Labs. Potassium chloride, calcium chloride, and sodium chloride powder were purchased from the Shanghai Institution of Biomedical Products.

### Experiments on marginal cells and 3T3 cells

#### Isolation, culture, and purification of marginal cells

Twenty neonatal rats were used for this experiment. Rats were euthanized using a rapid guillotine method, as approved by the Animal Care Committee. The stria vascularis and spiral ligament were dissected under a microscope (50×) and cut into 5 mm thick blocks. Blood cells were rinsed away using sterile saline solution. Tissue blocks were then cut into small pieces in sterile 1×PBS, and blood clots and connective tissue were carefully removed. A sterile 0.1% collagenase (type I) solution (w/vol; in serum-free 1640 culture medium) was used to dissociate the tissue into single cells for 4–6 hours in an incubator with 5% CO_2_ at 37°C. The single cells were examined under a microscope and were collected by centrifuge at 560×g for 3 minutes. The cells were rinsed once then resuspended in sterile 1×PBS and filter with a 200 mesh nylon membrane to remove. undigested fibers and connective tissue. Cells were collected with centrifuge and resuspended in 10 ml RPMI-1640 complete culture medium (containing 10% fetal bovine serum, 0.1% double antibotics; w/vol) and cultured in a 100 mm culture plate (5% CO_2_; 37°C). Dead cells and non-adhering cells were removed by refreshing the culture medium after a 12 hour culture. Adherent cells after 24 hour culture were tentatively identified as marginal cells of the stria vascularis of the cochlea. The culture medium was refreshed every 3 days. The cells were subcultured after 2 weeks of primary culture using the same medium. Cells at a concentration of 1×10^6^/ml were taken for identification.

#### Culture of 3T3 cells

The 3T3 cells were maintained in DMEM essential medium supplemented with 10% FBS vol/vol, and incubated at 37°C with 5% CO_2_. Cells were subcultured every 2 to 3 days. The same number of cells was used for the measurement as the marginal cells.

#### Verification of cultured marginal cells by flow cytometry

Marginal cells were dissociated with 2 ml of the 0.25% trypsin solution containing 0.02% EDTA for staining and counting with flow cytometry. The dissociated cells were incubated with anti-cytokeratin IgG (1∶500), anti-vimentin IgG (1∶500), or blocking soluting without antibodies for 2 hours at room temperature after 30 minute blocking, then followed by the FITC labeled secondary antibodies for 1 hour. The cells were washed three times after antibody incubation and collected by centrifugation and were then resuspended in 0.5 ml 1×PBS. The relative proportion of cells displaying fluorescence on their surface was determined with flow cytometry (Becton, Dickinson and Company, St. Franklin Lakes, NJ, U S A).

#### Fluorescence staining of vesicles containing ATP in cultured marginal cells

Marginal cell suspension aliquots were stained with quinacrine (1×10^−6^ mol/L; 1×PBS) for 20 min at room temperature in the dark and observed under a fluorescent microscope (Leica, Berlin, Germany). The control 3T3 cells were stained with same method as the marginal cells.

### Bioluminescence assay of ATP concentration in extracellular solution

#### Preparation of solutions

A CellTiter-Glo™ kit was used for these experiments in accordance with the manufacturer's instructions. In brief, the buffer solution was thawed to room temperature and then mixed in the brown bottle containing the substrate in equal volume. After gentle mixing, the substrate became completely dissolved within 1 min. This mixture was used as the reaction solution.

Stock solutions of reagents were serially diluted in multiproportion at different concentrations as needed for each experiment: Bafilomycin A1, 24–0.75 µM; DDA, 400–12.5 µM; KCl, 35.4–6.34 µM; thapsigargin, 1.6–0.05 µM; CaCl_2_, 5–0.31 mM; U73122, 8–0.25 µM; aristolochic acid, 400–12.5 µM. The calcium chelator EGTA (2 mM) was added to the extracellular fluid to titrate the extracellular Ca^2+^ concentration to 0 mM. This facilitated evaluation of the effects of the concentration of extracellular Ca^2+^ on the release of ATP from marginal cells.

#### Obtaining an ATP standard curve

An ATP stock solution (1 µM) was prepared and then used to deliver serial dilutions to aliquots of culture media. The reading plate was oscillated and incubated at room temperature for 10 min prior to reading. The fluorescent value of 5 serial concentration gradients of ATP solution was measured using a Glo-max TM 96 microplate luminometer (Promega) according to the manufacturer's instructions. Bioluminescence was read using a black 96-well plate to avoid optical cross-talk. The ATP standard curve was simultaneously measured using a 10-fold serial dilution of the ATP standard in each experiment. The standard curve of ATP was plotted using the concentrations of ATP solution and the fluorescent values. The concentration of ATP released from the test cells was calculated from the fluorescent value using the ATP standard curve.

#### Assay groups

Experimental groups consisted of cultured marginal cells treated with the testing reagents, including bafilomycin A1, DDA, KCl, thapsigargin, CaCl_2_, U73122, and aristolochic acid. The control groups were a suspension of marginal cells but without the addition of reagents, and 3T3 cells with testing reagents at their most effective dose. Each experiment were repeated 8 times. The average values were calculated.

#### Detection of ATP released from cultured marginal cells and 3T3 cells

Marginal cells or 3T3 cells in the culture flasks were inoculated into 96-well plates with 100 µl culture medium per well. Each well contained about 10,000 cells as counted using a cytometer. The background luminescence was also obtained by adding the same volume of culture medium, devoid of cells, to each well. One microliter of solution containing a testing reagent was added to a well. After 10 min of reaction at room temperature, 100 µl of fresh reaction solution was added to each well and mixed completely for 2 min. The plates were allowed to sit and incubate in the dark at room temperature for 10 min to stabilize the fluorescence signal prior to recording of luminescence. Then the fluorescence in each well was measured. ATP concentrations were calculated against the ATP standard curve. The fluorescence were detected 6 times and each experiment was repeated 8 time. The mean values were calculated. The results are expressed as mean ± SD.

### Statistical methods

Data regarding the average ATP concentrations of control and treated groups were analyzed by paired *t* test using SPSS 13. Software (SPSS Inc., Chicago, IL, U.S.). Results were considered significant at *P*<0.05.
