# Correction: Release of ATP from Marginal Cells in the Cochlea of Neonatal Rats Can Be Induced by Changes in Extracellular and Intracellular Ion Concentrations

**DOI:** 10.1371/annotation/7f401244-ab06-4243-8688-5c987ddca311

**Published:** 2012-10-23

**Authors:** Yating Peng, Jie Chen, Shan He, Jun Yang, Hao Wu

Yating Peng and Jie Chen are co-first authors. 

